# Mental practice promotes motor anticipation: evidence from skilled music performance

**DOI:** 10.3389/fnhum.2013.00451

**Published:** 2013-08-20

**Authors:** Nicolò F. Bernardi, Matteo De Buglio, Pietro D. Trimarchi, Alfonso Chielli, Emanuela Bricolo

**Affiliations:** ^1^Department of Psychology, University of Milano-BicoccaMilano, Italy; ^2^Music Conservatory “Giuseppe Verdi”Milano, Italy

**Keywords:** mental practice, motor anticipation, motor coordination, auditory imagery, motor imagery, music performance, musicians' injuries

## Abstract

Mental practice (MP) has been shown to improve movement accuracy and velocity, but it is not known whether MP can also optimize movement timing. We addressed this question by studying two groups of expert pianists who performed challenging music sequences after either MP or physical practice (PP). Performance and motion-capture data were collected along with responses to imagery questionnaires. The results showed that MP produced performance improvements, although to a lower degree than PP did. MP and PP induced changes in both movement velocity and movement timing, promoting the emergence of movement anticipatory patterns. Furthermore, motor imagery was associated with greater changes in movement velocity, while auditory imagery was associated with greater movement anticipation. Data from a control group that was not allowed to practice confirmed that the changes in accuracy and kinematics were not due to mere repetition of the sequence during testing. This study provides the first evidence of an anticipatory control following MP and extends the present knowledge on the effectiveness of MP to a task of unparalleled motor complexity. The practical implications of MP in the motor domain are discussed.

## Introduction

Mental practice (MP) has received substantial attention as a strategy for improving motor performance. MP is usually defined as the cognitive rehearsal of a task in the absence of overt physical movements (Driskell et al., 1994). Extended research in the field of sport psychology has shown that MP improves actual motor performance, although not to the same extent as physical practice (PP; Feltz and Landers, 1983). In both sport and music performance it has been evidenced that proper combinations of MP and PP yield results similar to extended PP alone (Kopiez, 1990; Theiler and Lippman, 1995; Bernardi et al., 2013). More recently, studies have been designed to understand the mechanisms that are responsible for these outcomes (Munzert et al., 2009). For example, in the music domain it has been shown that MP alone may lead to the same plastic changes in the motor system as those occurring with repeated PP (Pascual-Leone et al., 1995). For arm pointing and grapho-motor tasks, it has been observed that MP enhances movement speed (Yágüez et al., 1998; Gentili et al., 2010). No study to date has documented effects of MP on movement timing, which constitutes the focus of the present investigation.

Timing plays an essential role in skilled motor performance. Musicians anticipate the movements of specific body parts to ensure seamless execution of the forthcoming action (Engel et al., 1997; Baader et al., 2005). Expert athletes exhibit optimal movement timing, and coordinate the timing of different body parts more skillfully than beginners (e.g., Sanders, 1999). Interestingly, in several instances this coordination is achieved by decoupling the timing of different effectors. As one progresses in learning through PP, patterns of coordination are achieved in that the movement of one part of the body anticipates and prepares the movement of a different part, instead of having two effectors moving simultaneously (e.g., the vertical undulatory movement of the hip and knee during swimming, Sanders, 2007). These dynamics recall the process of coarticulation observed in speech (Ostry et al., 1996; Hardcastle and Hewlett, 1999) and fingerspelling (Jerde et al., 2003), in which the production of the current event is influenced by the upcoming events (anticipatory pattern). In all these tasks, timing is emphasized as a motor optimization parameter, allowing the seamless execution of an already well-learned sequence of movements.

However, timing has been shown to play an important role also when the exact order of the movements in the sequence is not acquired yet. For example, in serial reaction time tasks, participants are required to select and perform a response (e.g., moving toward a specific target) when a corresponding stimulus (e.g., the target becoming highlighted) is presented (Nakamura et al., 2001). Unbeknown to subjects, stimuli are presented in repeated sequences. As the sequence is presented over and over, participants show a decrease in their response time, assumed to represent the acquisition of the sequence (Nissen and Bullemer, 1987). Interestingly, the reduction of the time needed to produce the response is not necessarily dependent on explicit awareness of the sequence (Ghilardi et al., 2009). However, when the knowledge of the sequence becomes explicit, a qualitatively different phenomenon in movement timing appears, such that movement timing not only decreases, but also becomes *anticipatory* (defined as lower than simple reaction time floor value). The connection between movement anticipation and explicit awareness of the sequence has been shown in the form of a correlation between scores of declarative knowledge at the end of a serial reaction time task and the number of anticipatory movements (Ghilardi et al., 2003).

The present study aims to investigate whether MP is capable of influencing movement timing, when applied to learn a novel complex motor sequence. On the basis of the literature reviewed, we focused especially on two aspects of timing that have been shown to change following PP: movement anticipation and the relative coordination of effectors involved in producing the movement.

An effect of MP on the timing component that relates to motor optimization could be expected in the light of current models of motor control. According to the view of the *internal model* (Wolpert et al., 1995), both MP and PP utilize forward internal models: an efferent copy of the motor command is generated in the brain each time a movement is executed, regardless of whether the execution is actual or just imagined (Frith et al., 2000). This efferent signal would be used to make predictions about the future states of the effector. This model is useful to explain why the use of MP results in an increase of movement velocity (Gentili et al., 2006), similarly to what happens following PP. Given these assumptions, it is plausible to expect changes also in other dimensions of movement optimization, such as timing. However, contrary to PP, in MP the state estimates derive from the forward model alone, without any sensory feedback. This is advocated as the reason why, despite similar outcomes in terms of movement velocity, PP has been shown to achieve lower variability compared to MP (Gentili et al., 2010). The training signal in MP could therefore not be informative enough to faithfully represent the nuances required to fine-tune movement timing and inter-limb coordination, resulting in smaller and/or more variable changes, compared to PP.

Turning to the sequencing aspect of learning, a facilitation role of MP could be expected, with a positive impact on both movement accuracy and movement timing. It is known that MP is especially effective for the cognitive and strategic, rather than for the motoric components of a motor performance (Driskell et al., 1994). As discussed before, learning a sequence can rely on explicit memory and, when this happens, effects are to be expected especially on the timing dimension (Ghilardi et al., 2003).

Several aspects of previous research might have limited the possibility of detecting changes in movement timing and coordination following MP: (a) *Outcome measures as the focus of investigation*: in the sport and music psychology literature the main object of investigation is often the outcome of MP, measured as a task-specific dimension [e.g., distance between the ball stop point and the hole, for golf, Beilock and Gonso (2008); jumping height, for high jumping, Olsson et al. (2008); quality of performance assessed from expert raters, for music performance, (Theiler and Lippman, 1995)]. This limits the possibility to understand *how* the change in performance is actually achieved in terms of motor control. (b) *Task complexity*: in other fields of research, such as experimental psychology, extensive attention has been devoted to the mechanisms of MP, but simple tasks have mainly been adopted. As Gentili et al. (2006) noted, “previous investigations used relatively simple motor tasks that did not require high spatiotemporal or dynamic control of the action” (p. 761; see also Verstynen et al., 2012). In these simple motor tasks, fine motor adjustments are unlikely to be detected because they are not necessary and/or because they are already present at the baseline level. (c) *Ecological validity*: the notion of anticipation is partially related to the presence of an internal representation of the task. Tasks with poor ecological validity, such as those sometimes used in previous research, likely limit the possibility that changes in timing and coordination will occur. (d) *Subject selection*: It has been shown that wide differences exist within individual imagery abilities (Guillot et al., 2008), and that an adequate level of prior knowledge is required for MP to be effective (Finke, 1989); however, study participants are often not trained in MP and/or are not trained in the type of task employed. To overcome these possible limitations, the present study employed a piano playing task, as piano playing is known to involve highly complex sensorimotor control and a unique amount of independent motion of the fingers (Furuya et al., 2011). The subjects were all expert pianists, and each subject received explicit training in MP before the experimental session. The main goal was to assess whether movement anticipation dynamics, as well as changes in movement velocity and overall performance improvement could be detected after MP.

Additionally, we sought to identify associations between specific mental strategies and changes in different aspects of motor control. First, we expected changes in movement velocity to correlate with the use of motor imagery. Motor imagery has been previously shown to increase movement velocity in several tasks, from arm pointing (Gentili et al., 2006, 2010), to repetitive finger opposition (Avanzino et al., 2009) and circle drawing (Yágüez et al., 1998). In addition to the explanations in terms of forward internal models, a rationale for these findings relies in the observation that motor imagery increases corticospinal excitability of the muscle involved (Fadiga et al., 1999). The strength of corticospinal activation during motor imagery has been shown to be proportional to the vividness of imagery (Williams et al., 2012). Greater corticospinal excitability is likely to be accompanied by the possibility to move faster, as has been documented both in children during the course of development (Mueller and Hoemberg, 1992; Fietzek et al., 2000) and in adults (Koeneke et al., 2006). Second, we expected the emergence of movement anticipation to correlate with the use of auditory imagery. Although the connection between auditory imagery and motor performance have received less attention compared to motor imagery, there is evidence that in musicians auditory imagery enhances motor planning and results in earlier movement initiation. For example, Keller and Koch (2008) employed a response-effect compatibility paradigm and required people with various level of musical experience to produce auditory sequences by tapping on vertically aligned response keys. Assuming a conceptual correspondence between spatial height and pitch height, different mapping between keys and pitches were presented: in the high-compatibility condition, taps on the top, middle, and bottom keys triggered tones of high, medium, and low pitch, respectively. In the low-compatibility conditions this order was scrambled or reversed. Musical experience influenced the degree to which response initiation times were affected by the compatibility between movement trajectories and melodic contours on the vertical dimensions, with experienced musicians showing stronger effects of response-effect compatibility on response initiation time. The authors interpreted this result as an endogenous response priming by anticipated action-effects through auditory imagery. Auditory imagery would therefore allow the activation of anticipatory auditory-effect representations, resulting in priming of the appropriate motor response (see also Keller and Koch, 2006; Keller et al., 2010).

## Materials and methods

### Participants

Sixteen pianists (10 females) were recruited on a volunteer basis from local music conservatories. All participants gave their informed consent to the study. All participants had at least 9 years of individual piano instruction. The participants were randomly assigned to one of two groups: the MP group (*n* = 8) or the PP group (*n* = 8). The average total lifetime practice time was 17,006 ± 11,110 h for the MP group and 17,030 ± 6976 hours for the PP group. The average age was 30 ± 10 years for the MP group and 31 ± 9 years for the PP group. All subjects were currently performing piano at a professional level, and all gave their informed consent to participate in the study. All experiments reported in this study were in accordance with the ethical standards established in the 1964 Declaration of Helsinki.

### Preparation phase: MP training

Before the testing session, participants of both the MP and PP group individually underwent two 1-h MP training sessions. The first training session took place ~1 month before the test session. During the training session, the participants completed a preliminary self-report questionnaire assessing their familiarity with MP strategies. For each statement, they provided a score on a 1 (*never*) to 7 (*always*) Likert scale (e.g., “*When you study a piece of music that contains motorically challenging sequences, how often do you imagine the movements without actually moving?*”). After completing the questionnaire, the subjects followed a standardized training procedure derived from Klöppel's mental training manual (Klöppel, 2006). This procedure included (a) a concentration exercise focused on listening to the breath, (b) an exercise focused on the subject's own proprioceptive and somatosensory internal feelings, with a specific focus on the right hand, (c) reading and applying the instructions for learning to play a fast musical passage using MP. Exercises (a) and (b) were adapted from classic exercises used in mindfulness-based intervention and were administered by a psychologist trained in mindfulness intervention (author BNF). Exercise (c) was an adaptation of a detailed example from Klöppel's manual (2006, pp. 64–66). This exercise explained in details a step-by-step approach to the mental study of the first 4 bars of the *Finale* from the “Trio con pianoforte in Do min. op.1 n°3,” composed by Ludwing van Beethoven. Participants were provided with the music score of the excerpt. First, participants were instructed to focus on mental visualization, from an internal perspective. The exercise suggested to start with the visualization of the keys to press, and then turning to the visualization of the body postures and movements necessary to play these keys. Examples of instructions were: “*visualize as precisely as possible the keys on the keyboard corresponding to the written notes*,” “*visualize the position of the hand, the width of the movement of the arm*.” Participants were then invited to engage in the motor and auditory imagery of the passage. Motor and auditory images had to be produced together, throughout all the further stages. Initially a slow rendition was encouraged, breaking down the musical phrase and isolating its main components. These components were mentally rehearsed while progressively increasing the speed of the imagined performance. Examples of instructions were: “*Feel each single interval, in terms of both movement and sound, starting at a slow tempo*,” “*feel inside your body how the fingers should press the keys, initially using a legato touch*.” As participants progressed in the mental rehearsal, they were encouraged to occasionally try physically playing the passage practiced, and to switch between the imagined and the physical execution. The aim was to compare and refine their mental images with the information from the actual practice. Toward the end, participants were asked to introduce in the mental rehearsal the more challenging *fortissimo* dynamic and to abandon the legato touch. Having rehearsed the various small subcomponents, the instructions finally suggested to develop the mental execution of the sequence as a whole, integrated movement.

Following this first familiarization session, participants were asked to practice MP every day, applying it to the repertoire they were currently practicing, from the first day of training until the day of the testing session. In their daily practice, participants were advised to follow the aforementioned step-by-step procedure. However, they were also encouraged to adapt it to their own particular style and specific needs. Subjects received a diary for recording the daily time devoted to MP or PP. Diary reports confirmed that the subjects had completed daily MP exercises. The second training session took place ~2 weeks after the first and 2 weeks before the testing session. In this second training session, for familiarization purposes, the participants underwent a procedure identical to the one employed in the testing session (see section Procedure) to ensure that the subjects were not applying MP under unexpected conditions/requests on the day of testing. This training session differed from the testing session only in the musical piece chosen, which was Listz's Transcendental Etude No. 7. According to the local music school's teaching program, Listz's etude is considered significantly more difficult to play than the etude that was used for testing (see section Stimuli). This choice was made to ensure that well before the testing phase, all of the participants had tried at least once to apply MP to a level of musical motor complexity higher than the one that was actually tested in the experiment itself, thus limiting noise in the data due to possible disorientation. Listz's etude was not part of any of our subjects' repertoire, and only one subject in the sample had studied it in the past.

### Apparatus

The subjects were seated comfortably in front of a Roland RD-700 GX digital piano. The Roland RD-700 GX piano was connected to a computer, and MIDI data were recorded using SONAR LE software. Reflective markers were used to collect motion capture data with a three-dimensional optoelectronic movement analysis system (6 cameras, 120 Hz; SMART, BTS, Italy). For this purpose, three hemispherical markers with a 5-mm diameter were applied on the right hand to the (a) thumb fingernail, (b) little finger fingernail, and (c) styloid process of the ulna. All practice sessions and performances were video recorded using a digital video camera and showing the pianist from the front.

### Stimuli

**Figure 1:**
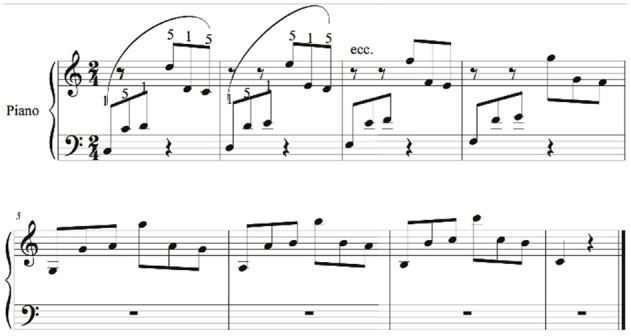
**Music piece.** The numbers over the notes represent the constrained fingering (1 = thumb, 5 = little finger). The piece had to be performed at a speed of 112 beats/min for each quarter note.

The arpeggio model from the first bar of Exercise 5a (WoO 6) for right hand only, from the 51 Exercises for piano composed by Johannes Brahms (1833–1897), was used in the testing session (Figure 1). We diatonically developed the model from C2 to C3 so that the total length of the exercise was 8 bars. To ensure that all of the pianists performed the piece the same way, the fingering was constrained so that for each bar, notes 1,3,5 had to be played with the thumb and notes 2,4,6 had to be played with the little finger. A pace of 112 beats per minute for the quarter note was set as the tempo, thus requiring pianists to play a note every 178 ms.

The following criteria lead to the choice of this piece for the present investigation: (a) It is essentially a motor-technical exercise, without specific musical character or expressive intent; as such, it does not lend itself to subjective spontaneous interpretations and tends to be performed consistently by different pianists with a neutral, “technical” character. (b) It is not included in any ordinary piano training program; this makes the piece unknown to the majority of pianists. (c) It is motorically challenging in terms of spatial accuracy (hitting the correct keys), timing (keeping up with the fast tempo) and coordination. (d) It is not *too* challenging, so that it can be roughly sight-read by any pianists with the minimal skill level we set; as such, it allows the recording of a meaningful baseline (see section Procedure). (e) It implies fine motor control of both the proximal (e.g., wrist) and distal (e.g., fingers) segments of the upper limb, thus allowing the study of different motor performance mechanisms. (f) It is suitable for a motion-capture setting; in fact, it does not require significant covering of the reflective markers and allows the gathering of the relevant information from a small set of markers.

### Procedure

**Figure 2:**
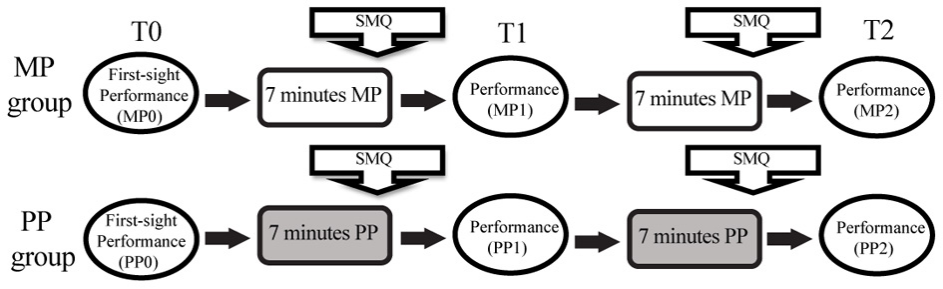
**Experimental paradigm.** MP, Mental practice; PP, Physical practice; SMQ, Seven-minute questionnaire aimed at describing the mental strategies used during practice.

Before the experiment started, all participants (a) familiarized themselves with the keyboard, (b) confirmed that the reflective markers applied to their right hand did not interfere with their playing, and (c) confirmed that they did not already know the piece. The procedure, summarized in Figure 2, began with the collection of a baseline performance (T0 = baseline; MP0, PP0) in which the subjects performed the Brahms exercise once by first-sight reading. The tempo was provided by a metronome for the duration of the performance. The subjects were explicitly asked (a) to use the written fingerings, (b) to play in synchrony with the metronome, (c) to perform the piece at *mezzoforte*, with regular force dynamics, (d) to perform the piece only once and from the beginning to the end, without interruptions or repetitions. The score was available to subjects from a few seconds before recording the baseline performance, and remained visible throughout the whole experiment.

The second phase differed for the subjects in the two groups. The subjects in the MP group received the following instructions: “*You have 7 min to practice this piece using MP and then you will perform it again. You can freely use whatever MP method you prefer, but you must avoid actual movements of your fingers/hands*.” During MP, the subjects had to position their right hands on the keyboard, which was fully covered by a rigid cover so that they could neither see nor feel the keys. Hand and finger stillness was monitored both visually and with continuous motion capture acquisition. After 7 min, the subjects completed a short questionnaire about the mental strategies they used during MP (see section Questionnaires) and then performed the piece at the keyboard (T1, MP1), following the same criteria as in the baseline performance. After this performance, the MP subjects had 7 more minutes to practice the same way as before; then they received another short questionnaire and gave a third performance (T2, MP2). Thus, the MP subjects had a total of 14 min of mental study interspersed with one performance. The duration of the entire session was calibrated according to the results of past research indicating that ~20 min is the optimal time for a MP session (Driskell et al., 1994). In contrast, the subjects in the PP group received the following instructions: “*You have 7 min to practice this piece and then you will perform it again. You can freely use whatever practice method you prefer, as long as it includes physically playing the instrument*.” The PP subjects were not asked to avoid or limit their mental operations during practice, as these constrains have been proven to be confounding and relatively ineffective (Bernardi et al., 2013). This choice designated our PP condition as the “natural,” ecologic practice condition and the one with which the subjects were more familiar. After the 7 min practice session, the subjects completed a short questionnaire about the mental strategies they used during PP and then performed the piece again (T1, PP1). Seven more minutes of PP were followed by the third and last performance (T2, PP2).

### Data analysis

#### Accuracy

Objective evaluations of performance accuracy were derived from the MIDI data. Two aspects of performance were evaluated: (1) spatial accuracy and (2) temporal precision. Spatial accuracy measures were obtained by counting the number of wrong notes. Temporal precision was expressed as a timing-error value. For each couple of notes, we calculated the difference between the prescribed inter-onset interval (178 ms) and the actual inter-onset interval that was performed. Error values were averaged across each performance, resulting in a single timing-error value (in ms) for each performance.

#### Movement kinematics

In the selected Brahms exercise, two repeated distinct phases involving only the right effector can be identified. The first phase, which we labeled the *Forward phase* (Frw), requires pianists to initially execute a fast wrist movement from left to right, followed by a rapid opening of the little finger relative to the thumb (notes 3–4) to catch the highest note. Following the Forward phase, the pattern is reversed, and the pianists must make a fast wrist movement from right to left, followed by a rapid opening of the thumb relative to the little finger (notes 6–7) to catch the lowest note; this sequence can be regarded as the *Backward phase* (Bck). This entire pattern is transposed identically for each of the seven notes of the C major scale, so that the exercise comprises 7 Frw and 7 Bck movements (14 movements overall). Critical aspects of performing the Brahms exercise are (a) the wrist movement and (b) the opening movement of the thumb and little finger. Therefore, the kinematic analyses focused on these aspects.

For each trial, kinematic data were analyzed offline for each of the 14 movements. The kinematic raw data were first filtered using a low-pass Gaussian smoothing filter with a sigma value of 0.93. Movement onset and offset were determined following the 5% peak velocity rule. The data were then averaged separately across all the Frw and Bck movements for each performance, resulting in a single mean and standard deviation value for the Frw movements and another mean and standard deviation value for the Bck movements. The following kinematic parameters were computed for the wrist marker (W) using the scalar value of the 3D velocity vector: (1) Wrist peak velocity (WPVel) and (2) Time to wrist peak velocity (TimeWPVel). Regarding the two finger markers (F), the following kinematic parameters were computed on the 3D distance between the thumb and little finger markers: (1) Finger opening peak velocity (FOpenPVel) and (2) Time to finger opening peak velocity (TimeFOpenPVel).

The pattern of coordination between the fingers and the wrist was assessed using the cross wavelet transform (for further details, see e.g., Torrence and Compo, 1998; Mallat, 1999; Grinsted et al., 2004), a time-frequency analysis method previously shown to be well-suited for the analysis of the interaction between two signals in human movement studies (Issartel et al., 2006). This method exposes regions with high common power spectra and reveals information about the phase relationship. Of particular interest for the present investigation was the phase angle between the wrist and finger movements. The cross wavelet transform was therefore computed between the wrist velocity and the velocity of the opening/closing of finger markers using the complex Morlet wavelet. The phase angle between the two signals was expressed in degrees as a value spanning from 180° (ϕ = π) to −180° (ϕ = −π). A phase angle of 0° represents perfectly in-phase relationships in which the wrist and finger peak velocities happen at the same time of the oscillatory dynamic. Phase angles progressively farther away from 0° represent an anti-phase pattern between the two oscillations, in which the movement in one effector happens while the movement in the other effector is still in preparation; angles of ±180° represent a perfect anti-phase pattern. An increase in the absolute value of the phase angle following practice was interpreted as an indicator of an anticipatory coordination pattern. Although the cross wavelet transform provides information about regions of high common power, it does not reveal information about the local correlation between the two time series in the time/frequency space. Therefore, to enhance the meaningfulness of the results, we first computed the wavelet coherence between the signals to detect locally phase-locked behavior, and we restricted the analysis of the phase angle to frequency bands showing a reliable level of coherence. We searched for the frequency bands in which the most statistically robust coherence could be detected (*p* < 0.05 along the entire time course of each recording and across the entire sample of 16 subjects). Only one frequency band showed overall reliable coherence (see section Movement coordination); therefore, subsequent analyses were focused only on this band. For each subject, the circular mean of the phase angles along the whole track was computed (see Zar, 1999 for the circular mean formula). To evaluate the variability of phase angle, we estimated the concentration parameter (*kappa*) of the Von Mises distribution (Mardia and Jupp, 2000) of phase angles along the entire track for each subject. Larger *kappa* values describe a distribution with a stronger concentration around the mean angle and therefore lower variability. The Matlab tools for analysis and the Montecarlo simulation provided by Grinsted (the functions *xwt*, *wtc, anglemean*; Grinsted et al., 2004; The MathWorks, Inc.) were used for these analyses.

### Questionnaires

Apart from overt movements, we deliberately did not constrain the MP subjects' strategies to allow the emergence of potential relationships between the individuals' choices and MP outcomes. The *Seven Minutes Questionnaire* (SMQ, Bernardi et al., 2013) was used to identify the different imagery modalities (auditory imagery, motor imagery, visual imagery, harmonic analysis) used during the practice session. This questionnaire has been validated and described in details in Bernardi et al. (2013). In addition to determine the imagery modalities used by the participants during the experiment, we sought to assess pre-existing individual differences in mental imagery, by means of the following standardized questionnaires: (a) the *USOIMM77* questionnaire, which assesses the spontaneous occurrence of mental visualization in thinking (Antonietti and Colombo, 1996); (b) the *Motor Imagery Questionnaire-Revised*, developed to examine kinesthetic and visual movement imagery ability (Hall and Martin, 1997); (c) the auditory subscale of the *Questionnaire of Mental Imagery*, which provides self-reported ratings of the vividness of auditory imagery (Olivetti-Belardinelli et al., 2009); (d) a non-self-report *Auditory Imagery Test* (Bernardi et al., 2013), requiring subjects to compare auditory presented pitches with written music notation. All these tests have described in details in Bernardi et al. (2013).

### Statistical analyses

Statistical analyses were conducted using SPSS 19.0. Repeated measures analyses of variance (ANOVA) with Time as a three-level within-subject factor (T0, T1, T2) and Practice as a two-level between-subjects factor (MP, PP) were conducted to assess changes in performance and movement kinematics. Movement variability was assessed using ANOVA on the standard deviation values of the velocity kinematic records. *Post-hoc* tests were computed using Sidak correction for multiple comparisons. Partial eta-squared (η^2^_*p*_) was assumed as a measure of effect size. Pearson's correlation coefficient (2-tailed) was employed to evaluate associations between changes in performance or kinematic parameters and the MP strategies used or the individual features quantified in the preliminary tests or questionnaires.

## Results

The MP and PP groups were homogeneous with respect to age and total lifetime practice time (independent *t*-test: both *p* > 0.05). All performance and kinematic parameters were tested for between-groups differences in the baseline, and an acceptable homogeneity of the MP and PP groups was confirmed (independent *t*-test: all *p* > 0.05). All variables showed normal distribution, as confirmed by the Kolmogorov–Smirnov test (all *p* > 0.05).

### Accuracy

**Figure 3:**
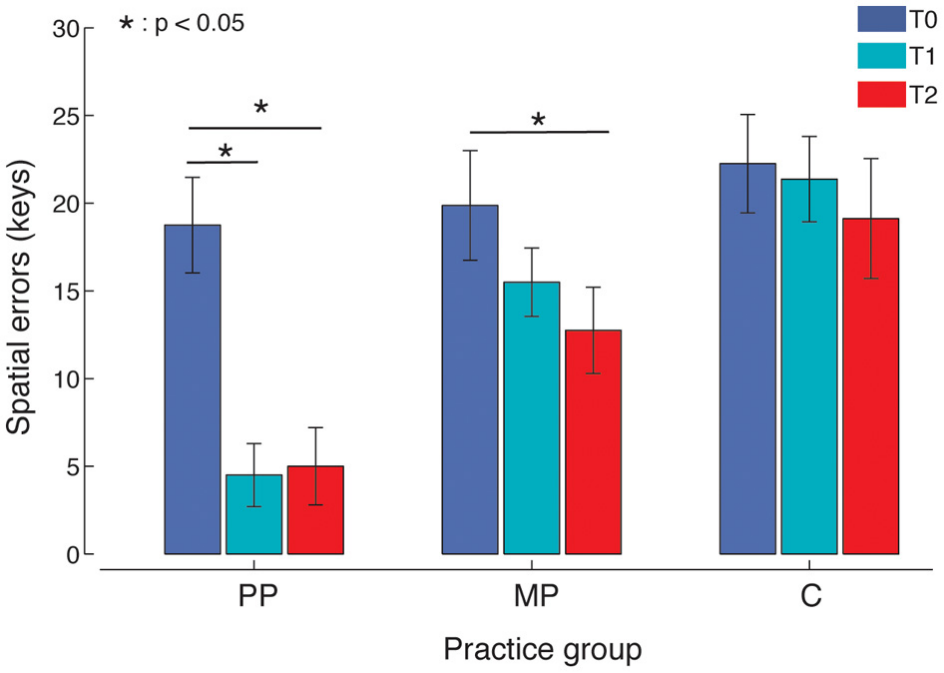
**Spatial errors.** PP, Physical practice; MP, Mental practice; C, Control group (no practice). Both MP and PP, but not C, significantly reduced the number of spatial errors. PP was effective after the first practice block and resulted in significantly better performance.

Both MP and PP improved movement accuracy, with PP yielding the strongest effect (Figure 3). An ANOVA of spatial errors showed a main effect of Time [*F*_(2, 15)_ = 23.96, *p* < 0.001, η^2^_*p*_ = 0.63, power = 1] and a significant Time × Practice interaction [*F*_(2, 15)_ = 4.62, *p* = 0.018, η^2^_*p*_ = 0.25, power = 0.73]. *Post-hoc* tests revealed that 14 min of MP produced a significant reduction in spatial errors compared to the baseline (*p* = 0.047). A significant improvement compared to the baseline was detected for PP at T1 (*p* = 0.001) and again at T2 (*p* < 0.001). PP resulted in fewer spatial errors compared to MP at both T1 (*p* = 0.001) and T2 (*p* = 0.034). Timing errors did not show any significant difference with respect to Time or Time × Practice interactions (*p* > 0.05).

### Movement kinematics

A summary of the kinematic data is reported in Table 1.

**Table 1:** **Summary of movement kinematics**.

	**MP**			**PP**		
	**T0**	**T1**	**T2**	**T0**	**T1**	**T2**
Time WPVel Frw (ms)^*^ ^†^	259 ± 20	256 ± 16	229 ± 17	258 ± 18	224 ± 8	218 ± 8
Time WPVel Bck (ms)^*^ ^†^	312 ± 45	244 ± 36	229 ± 34	268 ± 53	208 ± 28	183 ± 13
WPVel Frw (mm/s)^*^ ^†^	725 ± 38	762 ± 39	773 ± 34	771 ± 34	831 ± 23	863 ± 28
WPVel Bck (mm/s)^*^	614 ± 34	680 ± 41	713 ± 38	683 ± 39	675 ± 38	670 ± 39
FOpenPVel Frw (mm/s)^†^	998 ± 44	1118 ± 68	1001 ± 69	1170 ± 88	1340 ± 84	1363 ± 82
FOpenPVel Bck (mm/s)	801 ± 75	775 ± 70	765 ± 63	770 ± 54	849 ± 66	891 ± 63
Time WPVel Frw (SD), (ms)^†^	58 ± 12	56 ± 9	56 ± 11	52 ± 13	24 ± 3	21 ± 5
Time WPVel Bck (SD), (ms)	86 ± 29	53 ± 14	40 ± 11	28 ± 12	34 ± 11	23 ± 5
WPVel Frw (SD), (mm/s)	69 ± 9	92 ± 6	102 ± 13	76 ± 10	60 ± 6	49 ± 8
WPVel Bck (SD), (mm/s)	94 ± 11	77 ± 15	91 ± 17	77 ± 9	38 ± 4	37 ± 5
FOpenPVel Frw (SD), (mm/s)^†^	192 ± 30	164 ± 26	214 ± 39	213 ± 49	139 ± 32	122 ± 14
FOpenPVel Bck (SD), (mm/s)^*^ ^†^	149 ± 28	125 ± 18	130 ± 14	178 ± 30	89 ± 12	78 ± 6
Wrist-fingers phase (°)^*^ ^†^	100 ± 5	109 ± 4	108 ± 4	96 ± 5	106 ± 4	108 ± 3
Phase angle concentration^†^	10 ± 6	9 ± 7	13 ± 16	18 ± 16	80 ± 54	126 ± 106

*Data are reported as means ± standard error. MP, mental practice; PP, physical practice; T0, baseline performance; T1, performance following 7 min of practice; T2, performance following 14 min of practice; Frw, forward movements; Bck, backward movements; WPVel, wrist peak velocity; FOpenPVel, peak velocity of finger opening; SD, standard deviation*.

* *Significant changes (p < 0.05) were observed in the MP group between T0 and T2*.

† *Significant changes (p < 0.05) were observed in the PP group between T0 and T2*.

#### Movement timing

**Figure 4:**
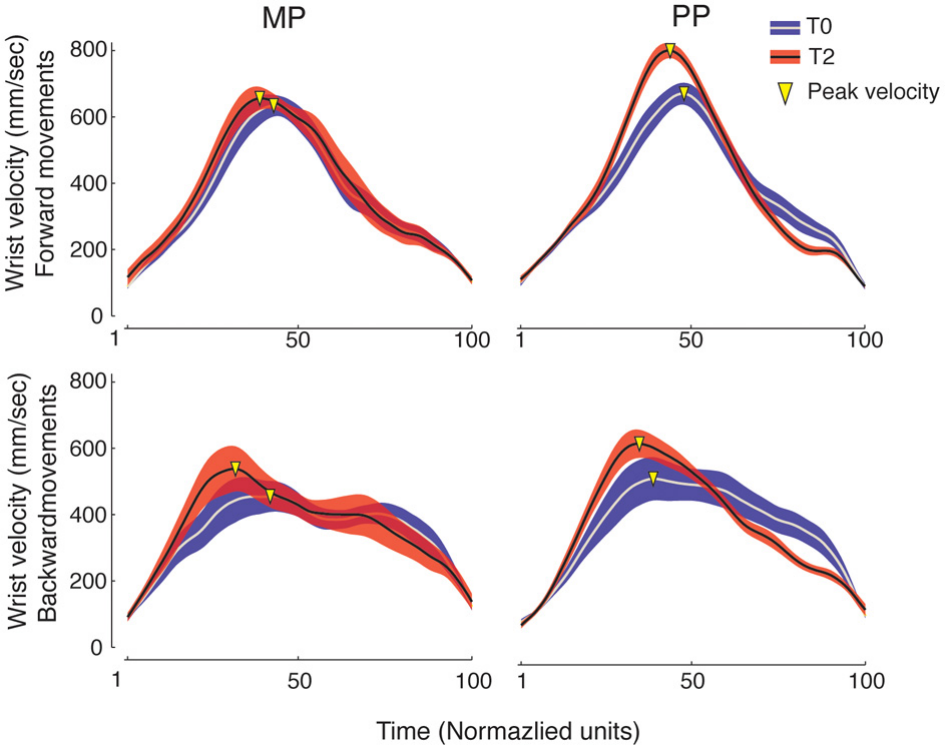
**Wrist velocity profiles.** Grand-averages (±SE) of wrist velocity profiles for the MP and PP groups (in each, *n* = 8). The time dimension was normalized to 100 units. Blue and red traces represent performance at baseline (T0) and following 14 min of practice (T2), respectively. Movement peak velocity is marked as a yellow triangle. It can be observed that at T2 the wrist peak velocity occurs earlier, indicating movement anticipation. This can be seen for both the MP and the PP group. Moreover, the peak velocity of wrist movements increases compared to the baseline.

Examples of wrist velocity profiles are shown in Figure 4. MP and PP similarly resulted in the anticipation of the peak velocity for the wrist movements. An ANOVA of the time to wrist peak velocity for the Frw movements showed a main effect of Time [*F*_(2, 15)_ = 13.15, *p* < 0.001, η^2^_*p*_ = 0.48, power = 0.99] and no significant Time × Practice interaction. The time to wrist peak velocity for the Frw movements decreased between T0 and T2 (*p* = 0.001) and between T1 and T2 (*p* = 0.008) with no significant differences between the two practice groups. An ANOVA of the time to wrist peak velocity for the Bck movements showed a main effect of Time [*F*_(2, 15)_ = 9.53, *p* = 0.001, η^2^_*p*_ = 0.41, power = 0.97] and no significant Time × Practice interaction. The time to wrist peak velocity for Bck movements decreased between T0 and T1 (*p* = 0.009) and between T0 and T2 (*p* = 0.022) with no significant differences between the two practice groups. No significant effects were detected in the time to peak velocity of finger opening for either the Frw or the Bck movements.

#### Movement velocity

MP and PP similarly resulted in an increased peak velocity for the wrist movements, but only PP also increased the velocity for the fingers movement. An ANOVA of wrist peak velocity for the Frw movements showed a main effect of Time [*F*_(2, 15)_ = 13.41, *p* < 0.001, η^2^_*p*_ = 0.49, power = 0.99] and no significant Time × Practice interaction. The wrist peak velocity of Frw movements increased from T0 to T1 (*p* = 0.038) and from T0 to T2 (*p* = 0.001) regardless of whether mental or PP was used. No differences were found between the MP and PP groups at either T1 or T2 (*p* > 0.05). An ANOVA of wrist peak velocity for the Bck movements showed a main effect of Time [*F*_(2, 15)_ = 3.72, *p* = 0.037, η^2^_*p*_ = 0.21, power = 0.63] and a significant Time × Practice interaction [*F*_(2, 15)_ = 6.32, *p* = 0.005, η^2^_*p*_ = 0.31, power = 0.86]. *Post-hoc* tests revealed that only MP increased wrist peak velocity, both from T0 to T1 (*p* = 0.046) and from T0 to T2 (*p* = 0.003); no significant changes were observed for the PP group. No differences were found between the MP and PP groups at either T1 or T2 (*p* > 0.05). An ANOVA of the velocity of finger opening for the Frw movements showed a main effect of Time [*F*_(2, 15)_ = 7.71, *p* = 0.002, η^2^_*p*_ = 0.36, power = 0.92] and a significant Time × Practice interaction [*F*_(2, 15)_ = 3.35, *p* = 0.05, η^2^_*p*_ = 0.19, power = 0.59]. *Post-hoc* tests revealed that only PP increased finger opening velocity from T0 to T1 (*p* = 0.013) and from T0 to T2 (*p* = 0.017). A difference between the PP and MP groups was found at T2, with PP subjects showing a higher peak velocity (*p* = 0.005). No significant effect of Time or Time × Practice interaction were detected for the velocity of finger opening for Bck movements.

#### Movement timing variability

A reduction in timing variability was observed for the PP group only. An ANOVA of the variability of time to wrist peak velocity for Frw movements revealed a main effect of Time [*F*_(2, 15)_ = 4.52, *p* = 0.02, η^2^_*p*_ = 0.24, power = 0.72] and a significant Time × Practice interaction [*F*_(2, 15)_ = 3.52, *p* = 0.04, η^2^_*p*_ = 0.2, power = 0.61]. A decrease in the variability of wrist timing was observed between T0 and T2 (*p* = 0.016). However, *post-hoc* tests on the interaction revealed that this effect was present only in the PP group (T0 vs. T2: *p* = 0.002); no changes were present in the MP group (*p* > 0.05). Moreover, the wrist timing of the PP group was significantly less variable than that of the MP group at both T1 (*p* = 0.008) and T2 (*p* = 0.013). No effects were detected in the variability of wrist movement timing for the Bck phase. No effects were observed in the variability of time to peak velocity of finger opening for either the Frw or Bck movements.

#### Movement velocity variability

Reductions in velocity variability were observed for both MP and PP, with PP yielding a stronger effect. An ANOVA of the variability of wrist peak velocity for Frw movements revealed a significant main effect of Practice [*F*_(2, 15)_ = 8.83, *p* = 0.01, η^2^_*p*_ = 0.39, power = 0.79]. *Post-hoc* tests showed that the PP group was significantly less variable than the MP group was at both T1 (*p* = 0.003) and T2 (*p* = 0.004). The variability of wrist peak velocity for Bck movements showed a significant main effect of Time [*F*_(2, 15)_ = 8.48, *p* = 0.001, η^2^_*p*_ = 0.38, power = 0.95] and no Time × Practice interaction; however, reliable differences could be found only between T0 and T1 (*p* = 0.004). The change between T0 and T2 was only marginally significant (*p* = 0.062), likely because of the high variability in the MP group. An ANOVA of the variability of the peak velocity of finger opening for Frw movements revealed a significant Time × Practice interaction [*F*_(2, 15)_ = 4.04, *p* = 0.029, η^2^_*p*_ = 0.22, power = 0.67]. *Post-hoc* tests showed that the PP subjects' variability decreased from T0 to T1 (*p* = 0.04) and from T0 to T2 (*p* = 0.034). Moreover, the PP subjects were significantly less variable than the MP group were at T2 (*p* = 0.044). An ANOVA of the variability of the peak velocity of finger opening for Bck movements revealed a main effect of Time [*F*_(2, 15)_ = 6.6, *p* = 0.004, η^2^_*p*_ = 0.32, power = 0.88]. This variability decreased from T0 to T2 (*p* = 0.043), and there were no significant differences between the two practice groups.

#### Movement coordination

**Figure 5:**
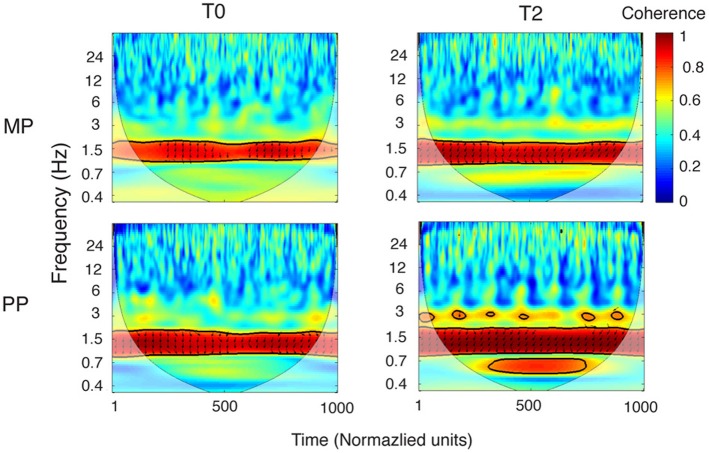
**Coherence between the wrist and finger velocity profiles.** Grand-averages of the coherence between the wrist and fingers velocity profiles, for the MP and PP group (in each, *n* = 8). Coherence is represented in the time-frequency space, with time normalized to 1000 units (the corresponding duration of performance was ~7.5 s). The cone of influence, where edge effects might distort the picture, is shown as a lighter shade. A high level of coherence indicates a systematically phase-locked behavior. The areas of statistically significant coherence (5% significance level against red noise) are shown surrounded by a thick black contour. It can be noticed that the wrist and the finger velocity profiles are reliably time-locked around a frequency of 1.5 Hz, consistently with the movement speed required to perform the exercise. Wrist-fingers coherence around 1.5 Hz is already present at baseline, and becomes stronger as a result of practice. This change is more pronounced following PP, compared to MP. The arrows depict the information about relative phase relationship, with in-phase arrows pointing toward the right and anti-phase arrows pointing toward left. From T0 to T2 the phase relationship tends to shift toward a stronger anti-phase pattern (that is, more arrows pointing leftward; see also Figure 6), for both MP and PP.

Coherence analysis was performed on the entire movement recording. Testing the reliability of the coherence between the wrist and finger velocity profiles revealed a consistent statistical significance for the frequency of 1.53 Hz (Figure 5). At this frequency, the coherence was significant (*p* < 0.05) at every single time point in 89% of the recordings (43/48). The five exceptions were two MP0, two MP1, and one MP2 recordings. Significant coherence at 1.53 Hz in these five performances was maintained for 31.4 and 87.5% of the timeline in the two MP0 files, for 85.04 and 85.6% in the two MP1 files and for 80.9% in the MP2 file. The frequency of 1.53 Hz describes events happening approximately every 650 ms. Interestingly, this value is close to the prescribed periodicity of the Frw and Bck phases. In fact, both the Frw and Bck phases consist of three notes, each with a prescribed duration of 178 ms, so that 178 ms × 3 = 534 ms.

**Figure 6:**
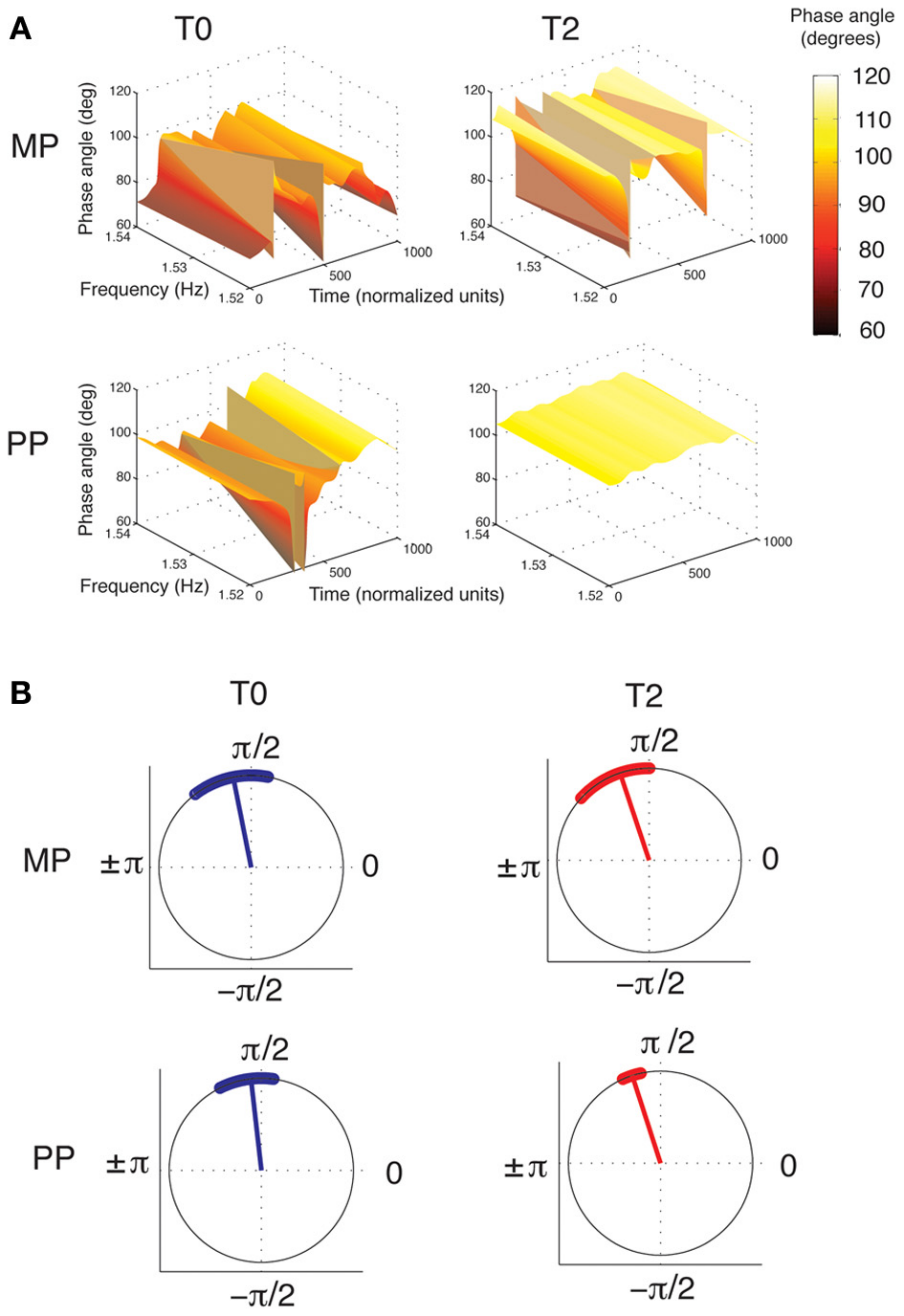
**Phase relationship between the wrist and fingers velocity profiles. (A)** Grand-averages of the phase angles between the wrist and the fingers velocity profiles, for the MP and PP group (in each, *n* = 8). Phase angles are represented in the time-frequency space. Time was normalized to 1000 units (the corresponding duration of performance was ~7.5 s). The frequency axis is zoomed-in around the value of 1.53 Hz, where the wrist-fingers coherence was found to be maximal (see Figure 5), and therefore the phase information more reliable. It can be seen that from T0 to T2, the overall phase angle increases. This represents a shift toward a stronger anti-phase pattern of coordination, in which peak velocity is achieved by one effector while the other is preparing the successive movement. MP and PP produces similar changes in the overall pattern, but PP results in greater stability of the phase angles across the time dimension. **(B)** Circular plot of phase angles between the wrist and finger velocity profiles. The dots along the circumference represent the distribution of phase angles during performance averaged across all subjects at each time point (for the purpose of this plot, the time has been normalized to 1000 points). The radius represents the circular mean of these angles. For both MP and PP, the phase angle increases from T0 to T2. However, only after PP does the distribution of phase angle become narrower, implying greater consistency of the phase angle during performance. Circular plots were created using the scripts provided by Berens (2009).

An ANOVA of the phase angle between the wrist and finger velocity profiles at 1.53 Hz, averaged across the whole performance, revealed a main effect of Time [*F*_(2, 15)_ = 11.88, *p* < 0.001, η^2^_*p*_ = 0.46, power = 0.99]. For both the MP and the PP groups, the phase angle increased from T0 to T1 (*p* = 0.006) and from T0 to T2 (*p* = 0.003; see Figure 6A). This means that with practice, the wrist and finger velocity profiles moved toward a stronger anti-phase pattern of reciprocal coordination. No differences were found between the two groups at any time point. Similar results were found when the phase angles were averaged separately for the Frw and the Bck movements. However, a clear superiority of PP compared to MP emerged when comparing the variability of the phase angle throughout the performance. The ANOVA of the variability of phase angle revealed a main effect of Time [*F*_(2, 15)_ = 5.62, *p* = 0.009, η^2^_*p*_ = 0.29, power = 0.82] and a Time × Practice interaction [*F*_(2, 15)_ = 5.1, *p* = 0.013, η^2^_*p*_ = 0.27, power = 0.78]. The phase angle variability significantly decreased (that is, the concentration increased) from T0 to T1 (*p* = 0.016) and from T0 to T2 (*p* = 0.044). A significant decrease in variability with time was observed for the PP group only (for PP, T0 vs. T1: *p* = 0.001; T0 vs. T2: *p* = 0.006; for MP, all *p* > 0.05). Despite the two groups' similar variability at T0 (*p* > 0.05), the PP group showed lower variability than the MP group did at both T1 (*p* = 0.003) and at T2 (*p* = 0.01). Figure 6B shows the grand-averages of phase angle distributions for the two groups.

### MP: strategies and outcomes

**Figure 7:**
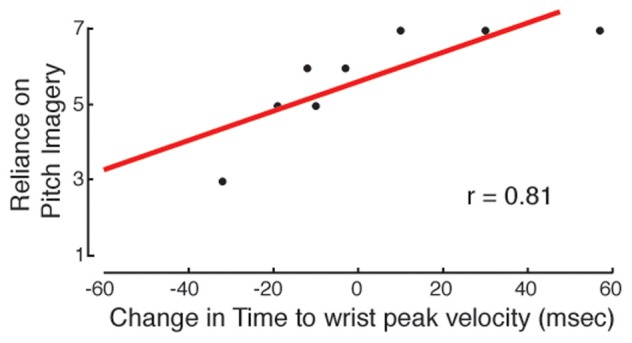
**Auditory imagery and movement anticipation.** In the MP group, the development of movement anticipation from MP0 to MP1 (measured as the difference in time to wrist peak velocity) is related to the use of auditory imagery (pitch imagery). The more the subjects reported having used auditory imagery, the more they showed anticipation of wrist peak velocity.

The preliminary questionnaire administered in the first training session was used to infer the participants' long-term developed habits of musical MP. Information about the practice strategies that were actually applied during the experiment was derived from the two SMQ questionnaires. Because our aim was to investigate relationships between the questionnaire's scores and the effectiveness of practice, correlations were not based on the raw performance or kinematic data. Instead, for each performance and kinematic parameter that showed significant improvement, we computed differential scores that expressed the *change* in the values (a) in the early phase of practice, from T0 to T1 (Diff1 = MP1 − MP0) and (b) across the whole practice session, from T0 to T2 (Diff2 = MP2 − MP0). Regarding the early phase of practice, significant correlations were found for motor imagery and auditory imagery. Subjects who were more familiar with motor imagery exhibited a greater enhancement of wrist peak velocity, as shown by the correlation between the Diff1 scores for wrist peak velocity and the habit of relying on motor imagery, as reported in the preliminary questionnaire (Pearson's correlation coefficient, 2-tailed: *r* = −0.73, *p* = 0.043). The correlation between Diff1 scores for wrist peak velocity and the actual use of motor imagery, as reported in the first SMQ, was marginally significant (*r* = −0.69, *p* = 0.058). Regarding pitch imagery, the use of this strategy during MP was connected to successful movement anticipation (Figure 7): Diff1 scores for the time to wrist peak velocity, for Frw movements, were significantly correlated with self-reports of pitch imagery use from the first SMQ (*r* = 0.81, *p* = 0.015).

When the practice session was considered as a whole, two other associations could be detected. A decrease in the number of wrong notes was associated with the habit of relying on external auditory models (e.g., recordings of experts' performances), as reported in the preliminary questionnaire. Subjects with a more established auditory modeling habit achieved greater spatial accuracy improvement after 14 min of MP (*r* = −0.725, *p* = 0.042). In comparison, subjects who more frequently engaged in harmonic analysis of the piece throughout the MP session (SMQ1 + SMQ2 scores for Harmonic analysis) showed smaller increases in wrist peak velocity (*r* = 0.78, *p* = 0.023). No associations were observed between practice outcomes and individual differences in the use of different imagery formats, as reported in the USOIMM77, Motor Imagery Questionnaire-Revised, self-report and non self-report auditory imagery tests.

### Control experiment

The data presented above showed an effect of MP on movement accuracy and kinematics. However, it cannot be excluded that these effects were solely due to the fact that at T1 and T2 pianists were performing the task for the second and third time. We therefore studied an additional group of pianists (*n* = 8; 1 female; age = 32 ± 9 years; total lifetime practice = 17,020 ± 11,016 h) to serve as a control group (group C). These subjects received the same training in MP as described in section Preparation phase: MP training. On the day of testing, they first-sight played the exercise (C0) and performed the piece again after 7 (C1) and after 14 (C2) min. However, in the interval between the performances, they were not allowed to practice the piece. Instead, they were engaged in filling the same questionnaires about mental imagery that were administered to the other subjects after the last performance. The music score of the exercise was not visible during the questionnaire filling, but was available during performance. The mental operations required from these subjects in order to fill in the questionnaires were in general very similar to those used during MP (e.g., motor, auditory, visual imagery), but for control subjects these operations were not focused on practicing the piece. Significant changes were found for the variability of wrist timing in the backward movement [*F*_(1, 7)_ = 8.78, *p* = 0.003, η^2^_*p*_ = 0.56, power = 0.93] and for the velocity of finger opening in the forward movement [*F*_(1, 7)_ = 4.31, *p* = 0.035, η^2^_*p*_ = 0.38, power = 0.65]. *Post-hoc* tests showed that the variability of wrist timing in the backward movement decreased from T0 (95 ± 36 ms) to T1 (36 ± 20 ms; *p* = 0.0002). However, no differences were found between T0 and T2 (65 ± 50 ms, *p* = 0.4) or between T1 and T2 (*p* = 0.13). Regarding the velocity of finger opening in the forward movement, *post-hoc* tests showed an increase from T0 (1044 ± 271 mm/s) to T1 (1178 ± 303 mm/s; *p* = 0.009). However, no differences were found between T0 and T2 (1198 ± 425 mm/s, *p* = 0.16) or between T1 and T2 (*p* = 0.99). No significant differences were found in any other kinematic or accuracy measure.

## Discussion

The present investigation has gathered the first evidence that MP results in movement anticipation. Movement anticipation has been documented here in two ways: first, as an earlier occurrence of the peak of movement velocity; second, as a change in the relative coordination of the two effectors involved in the movement (i.e., wrist and fingers). In fact, MP promotes a shift from a pattern of co-occurrence of the two oscillatory dynamics to a pattern of alternation. These changes mirror those following PP, but only PP also reduced movement variability. In addition, we confirmed previous results showing that MP produces significant performance improvements and increases movement velocity on a highly skilled motor task. Finally, we documented associations between specific components of MP (motor imagery, auditory imagery) and changes in different aspects of motor control (speed, anticipation).

### Mechanisms of mental and physical practice

A number of processes are involved when learning a new motor sequence (Hikosaka et al., 2002). First, one has to memorize the order of the elements in the sequence. Studies employing serial reaction time tasks have shown that, through practice, an initially unknown sequence becomes progressively familiar. Accordingly, a reduction of errors in the selection of the correct item of the sequence is observed (Nakamura et al., 2001). Practicing the sequence through actual movements is regarded as the most effective way to accomplish learning. However, MP could also be effectively used to rehearse the sequence and to strengthen its mental representation (Jeffrey, 1976). Accordingly, in the present study both MP and PP improved movement accuracy. Previous studies also established that the effectiveness of MP decreases as the task involves purely motoric, rather than cognitive/strategic, components (Driskell et al., 1994). In line with these findings, we found that PP was more effective than MP, as the present design emphasized the motoric dimension of performance. With this respect, it is interesting to notice the difference in the learning curves following MP and PP, with PP resulting in an exponential-like decrease in the number of errors, and MP resulting in a slower and constant improvement, resembling a linear trend.

Acquiring the sequence order is just a first step in developing a skilled performance. Especially when fine motor control is involved, a necessary second step involves optimizing the execution of each element in the sequence (Penhune and Steele, 2012). Movement timing and movement velocity are two aspects of skilled motor performance that PP has been shown to optimize (e.g., Sanders, 1999; Khlifa et al., 2013). A surprisingly small number of studies has investigated whether this is the case for MP also, as most investigations have been concerned with practice outcome alone. A consistent report in these selected studies is an increase in movement velocity following MP that closely resembles the effect of PP (Yágüez et al., 1998; Gentili et al., 2006, 2010). The present study confirms these previous findings, showing an absolute increase in movement peak velocity following both MP and PP. This observation is complemented here by the novel finding that, through MP, movements not only become faster, but are also executed *earlier*. Interestingly, this effect is found only on the relatively more proximal effector (i.e., the wrist), and not on the distal effector that is directly engaged in the production of the sounds (i.e., the fingers). This suggests that movement anticipation is employed as a strategy to pre-arrange the hand in a strategic position, allowing a more comfortable execution of the finger movements that follows. On the other hand, the timing of finger movements is not modified. This is understandable given the strict time constraint that the fingers have to obey to reach each key at the right time.

A further aspect of movement optimization pertains to the temporal coordination between the various effectors involved in the movements. For example, finely timed coordination is required to achieve fluent sequence production in coarticulation (Hardcastle and Hewlett, 1999), and/or to maximize movement efficiency in the execution of multi-limb complex actions (e.g., by reducing muscle-dependent torques, Furuya and Kinoshita, 2007). The present study has shown for the first time that MP is similar to PP in promoting an anti-phase pattern of inter-limb coordination. The utility of these patterns is understood, for example, in terms of maximizing movement smoothness and assisting in energy conservation (Sanders et al., 1995).

While MP and PP appeared to have similar influences on movement velocity, timing, and coordination, this was not the case for the variability associated with each of these dimensions. In most cases, PP was shown to result in more stable and reliable movement kinematics. These results expand and confirm those from previous investigations (e.g., Papaxanthis et al., 2002), and fit with the prediction of current theories of motor control (Wolpert et al., 1995). The use of the forward internal models would allow the prediction of the future sensorimotor state of the limb based on both its current state and the efferent copy of the motor command (Gentili et al., 2006). To the degree that this estimate is accurate, training through the forward model alone can refine future motor commands and lead to effective plastic neural changes (Desmurget and Grafton, 2000). However, because in MP the sensory feedback is absent, a great margin for variability is created. Consistently with this idea, as soon as some feedback is provided through minimal PP, the learning curve following MP shows sudden accelerations (Pascual-Leone et al., 1995; Bernardi et al., 2013), greater than what one would expect from PP alone given the little amount of practice.

In principle, the anticipatory pattern we have documented could be related not only to motor optimization, but also to the preliminary learning of the order of the elements in the sequence. In fact, timing has been regarded both as a sequencing (Ghilardi et al., 2003, 2009) and as an optimization parameter (Penhune and Steele, 2012). In the present design it is possible that both aspects of learning acted in synergy to promote the anticipation of wrist movements. However, given the motoric emphasis of the task we employed, we believe that the effects we have described mainly reflect a process of motor optimization.

A bias in the interpretation of the outcome of MP could be introduced by the fact that subjects performed the task three times during our experiment. This mere repetition could have alone allowed performance improvements, regardless of the practice content. However, the data from the control group we provided suggest that this is not the case. Subjects that did not engage in any practice did not show any improvement in accuracy. In these subjects, isolate changes in movement kinematics appeared to be unstable, being found at the first repetition, but not in the second. Moreover, none of these changes were related to movement anticipation and coordination.

### Mental practice strategies

It is known that several imagery modalities and mental strategies can be employed in MP (Roeckelein, 2004). The dominant focus of past research has been motor imagery, which is reasonable considering that the desired output of practice is in most cases a motor response. However, it appears that the role of complementary sensory modalities has often been neglected; moreover, the view of motor imagery as an exclusive force and effort has been questioned by authors who propose that motor imagery might also include visual and/or spatial components (Smyth and Waller, 1998; Callow and Hardy, 2004). Music performance offers an example in which this issue is particularly evident, given the tight coupling between the motor, auditory, somatosensory, and even visual representations of performance movements (Lotze et al., 2003; Haslinger et al., 2005). Systematic investigations of the effectiveness of different MP patterns are scarce. In a recent study involving the memorization of a long sonata that was musically complex but motorically easy, Bernardi et al. (2013) found that optimal memorization was achieved by subjects who (i) had a stronger habit of formal/structural analysis and (ii) particularly engaged pitch imagery during MP. The present results offer a complementary picture. In a task involving the score-supported performance of a short music exercise with minimal musical content but high motor complexity, we found that (i) motor imagery was associated with increased movement velocity; (ii) formal/structural analysis appeared to have a detrimental effect on movement velocity; and (iii) auditory imagery was associated with greater movement anticipation. The first finding directly supports the idea that motor imagery might improve motor control through the efferent copy of the motor command, as previously discussed. This result is also in line with the finding that corticospinal facilitation during motor imagery is associated with ease of kinesthetic imagery in both expert athletes (Fourkas et al., 2008) and in the general population (Williams et al., 2012). The second finding underlies the specificity of the linkage between motor imagery and state estimation improvement: a generic understanding of the piece's structure at a conceptual level alone does not lead to increased movement velocity; furthermore, it might even have detrimental effects when, in a limited time-window, it takes resources away from the motor focus. Regarding the third finding, an association between auditory imagery and movement anticipation within a musical sequence has been described in several studies (Keller and Koch, 2006, 2008; Keller et al., 2010). Keller and Koch (2006, 2008) have argued that auditory imagery might enable rapid and thorough action preplanning via an ideomotor-like mechanism in which actions are triggered and facilitated by the imaginary anticipation of their effects (Hommel et al., 2001; Knuf et al., 2001). The results presented here provide converging evidence for this idea, showing for the first time the emergence of movement anticipation as related to, and possibly as a result of, auditory imagery of the sequence during MP. When these and past results are considered together, auditory imagery emerges as the operational core of MP in the music domain (see also Highben and Palmer, 2004; Brown and Palmer, 2013), subserving both the construction of a structural/conceptual representation of the piece and the motor implementation of the precise movements. In this respect, it is interesting to notice how auditory cortical areas can in fact be recruited during auditory imagery, even in the absence of sound (Zatorre and Halpern, 2005). In addition, both the present study and the previous Bernardi et al. (2013) study showed how a general habit of auditory modeling (e.g., listening to expert performances as a way to improve one's own performance) tends to improve MP's effectiveness. The role of other strategies may vary depending on the specific task, with formal analysis and motor imagery being relevant for memorization and motor optimization, respectively.

### Practical implications

The present study provides implications for the applied use of MP. First, these results have a straightforward application to musicians' training and the management of health-risk factors. Overuse injuries are the leading cause of playing-related medical problems, and in some cases, such injuries can threaten or end a musician's career (Lockwood, 1989). Previous studies have described the effectiveness of MP in several aspects of music performance (Coffman, 1990; Kopiez, 1990; Theiler and Lippman, 1995; Cahn, 2008), and this study has gathered the first evidence that MP can also be effectively used to rehearse complex motor sequences in the music domain. Fine motor skills practice is crucial for musicians, and most of music students' time is devoted to such practice; therefore, it is potentially more connected to playing-related illnesses. Musicians' practice habits could therefore be enriched by combining their PP with MP, allowing performance improvements without any further cost to the body. Second, MP is increasingly being considered as a potential tool for motor rehabilitation in stroke patients (Lotze and Cohen, 2006; Barclay-Goddard et al., 2011). With respect to this application, this study suggests that fine motor skills, involving control of different effectors, could be addressed in the future. This study also suggests that the independent control and coordination/coarticulation of different effectors could be informative of the outcome of MP in movement rehabilitation, in addition to the more standard measures of, for example, velocity and force.

The main limitations of the present study pertain to the small sample size and the variability in the degree of music experience and motor performance between the subjects examined. Larger and more homogeneous samples, yielding stronger statistical power, will be necessary to directly compare kinematic and performance outcomes between different practices and with no-practice conditions. Another interesting advance to the present design would be to employ a natural piano with embedded digital recording (e.g., Disklavier™ piano), to study subtler aspects in the control of the touch and of the musical dynamics. Finally, it will be interesting to study the effect of MP on motor control when applied to musical excerpts with greater structural and technical complexity.

In conclusion, this study has shown how MP can improve fine motor control in terms of movement velocity, movement timing, and coordination. Future investigations should address how these processes are handled at the level of the neural bases and to what extent the effects described here are linked to partially distinct brain circuitries.

### Conflict of interest statement

The authors declare that the research was conducted in the absence of any commercial or financial relationships that could be construed as a potential conflict of interest.
